# Modulating the cobalt dose range to manipulate multisystem cooperation in bone environment: a strategy to resolve the controversies about cobalt use for orthopedic applications

**DOI:** 10.7150/thno.37931

**Published:** 2020-01-01

**Authors:** Guanqi Liu, Xiaoshuang Wang, Xuan Zhou, Linjun Zhang, Jiaomei Mi, Zhengjie Shan, Baoxin Huang, Zhuofan Chen, Zetao Chen

**Affiliations:** 1 Institute of Stomatology and Department of Oral Implantology, Guanghua School of Stomatology, Hospital of Stomatology, Sun Yat-sen University and Guangdong Provincial Key Laboratory of Stomatology, Guangzhou 510055, China;; 2 Zhujiang New Town Clinic, Guanghua School of Stomatology, Hospital of Stomatology, Sun Yat-sen University and Guangdong Provincial Key Laboratory of Stomatology, Guangzhou 510055, China.

**Keywords:** cobalt, systems immunology, osteoimmunomodulation, angiogenesis, bone regeneration.

## Abstract

The paradoxical effect of cobalt on biological processes has aroused controversy regarding the application of cobalt-based biomaterials in bone regeneration. Tuning the dose range of cobalt ions may be a valid strategy to resolve the controversies about cobalt use for orthopedic applications. Recent progress in bone biology has highlighted the effects of multisystem cooperation (especially of osteoimmune, skeletal, and vascular systems) on bone dynamics. Before the application of this dose-tuning strategy, a deeper understanding of its dose-dependent effect on the cooperation of osteoimmune, skeletal, and vascular systems is needed. However, due to the difficulties with investigating the interaction of multiple systems *in vitro*, the multimodal effects of cobalt on bone homeostasis were investigated here, in an *in vivo* scenario.

**Methods**: *In vitro* CCK8 assay and cytoskeletal staining were preformed to detecte the cell cytotoxic reaction in response to 0.1-100 ppm cobalt stimulation. Blood clot containing 0.1 to 5 ppm of cobalt were implanted in the rat calvarium defect. The gene profile of osteoimmune, skeletal, and vascular system as well as the systemic toxicity were evaluated *via* RT-qPCR, histological analysis and inductively coupled plasma mass spectrometry. The bone regeneration, osteoclastogenesis and vascularization were assessed by micro-ct and histological analysis.

**Results**: Cobalt concentration below 5 ppm did not cause cell toxicity* in vitro.* No systemic toxicity was observed* in vivo* at 0.1-5 ppm cobalt concentration. It was found that the early cytokine profiles of the multiple interacting systems were different in response to different cobalt doses. Most of the anti-inflammatory, osteogenic, and proangiogenic factors were upregulated in the 1 ppm cobalt group at the early stage. In the late stage, the 1ppm group was most superior in bone regenerative effect while the 5 ppm group displayed the strongest osteoclastogenesis activity.

**Conclusions**: The 1 ppm concentration of cobalt yielded the most favorable cooperation of the osteoimmune, skeletal, and vascular systems and subsequently optimal bone regeneration outcomes. Tuning the cobalt dose range to manipulate the cooperation of osteoimmune, skeletal, and vascular systems could be a promising and valuable strategy to prevent paradoxical effects of cobalt while preserving its beneficial effects.

## Introduction

Cobalt-based alloys, because of their excellent mechanical properties, have been used in hip and knee replacements, with some clinical success 1, 2. Nonetheless, the long-term performance of the cobalt-based orthopedic implants in patients is unsatisfactory due to the adverse biological reactions caused by excessive generation of cobalt particles and ions 3-6. High levels of cobalt ions in the body may cause an excessive inflammatory reaction, osteolysis, and consequently failure of the implant 2, 7. Meanwhile, many studies have shown that cobalt is a toxic agent that may cause an allergic reaction and tissue destruction 8-10. As a result, the use of cobalt-based implants has been reduced, and they have been ousted by other types of orthopedic implants.

The importance of vascularization in bone regeneration has been well illustrated 11. Blood vessels deliver nutrients, oxygen, and growth factors, which are vital for bone repair. However, achieving vascularized bone regeneration has been challenging. Angiogenesis-targeted bone-regenerative strategies have been investigated such as bone grafting with a vascular bone flap, gene therapy with nucleic acids encoding proangiogenic vascular endothelial growth factor (VEGF) 12, 13, and application of expensive recombinant VEGF proteins 14, 15. However, the application of these approaches is limited by such disadvantages as the additional injury caused at the donor site 16, 17, the high dose of a protein required, and low transfection efficiency associated with gene therapy 18. Thus, efforts have been devoted to the exploration of alternative strategies. One of the significant successes is the discovery that the cobalt ion has a positive effect on vessel formation and enables the coupling of osteogenesis and angiogenesis by stabilizing the hypoxia-inducible factors (HIFs); this event initiates a cascade of proangiogenic factors including VEGF 17, 19. Being inexpensive, potentially easy to operate, and having favorable effects on osteogenesis and angiogenesis, cobalt as a promising tissue regeneration tool has been receiving attention in the bone regeneration field. Cobalt-induced vascularized bone regeneration has been developing into a subdiscipline 17. It follows the notion that cobalt does not have only adverse biological effects. Minimizing the detrimental effects and making good use of the favorable properties of cobalt may be a practicable strategy to develop cobalt-based regenerative biomaterials.

After reviewing studies related to biological effects of cobalt-based biomaterials, we found that the bone-regenerative ability, immune response, and angiogenic reaction are different across studies due to the diverse concentrations of the released cobalt ions 17, 20, 21. The release of cobalt ions from the cobalt bioactive glass/collagen-glycosaminoglycan composites ranges from 3 to 12 ppm after 24 h and 7 days 17. The cobalt concentration of cobalt mesoporous bioactive glass-soaked media has been reported to be 20 ppm at 7 days 20. Core-shell hydrogel scaffold prepared with different cobalt concentration solutions released cobalt ion from 0.015 mM to 6 mM at 6 days 22. In some cobalt-based materials, only a small amount of cobalt ions is released. The levels of cobalt ions from cobalt-doped hydroxyapatites have been reported to be 0.002 to 0.136 ppm at 60 days 21. With different doses of cobalt loaded, the release of cobalt ion form cobalt-doped hydroxyapatite ranges from 0.083 to 0.247 ppm at 60 days 23. The concentration of cobalt ions detected in the extracts of cobalt-incorporated tricalcium phosphate is 0.0015 to 0.0118 ppm 24. 2% cobalt-incorporated β-tricalcium phosphate improved bone formation while excessive Co doping decreased tricalcium phosphate induced osteogenesis *in vitro* and *in vivo* 25. It seems that different osteogenic activities, angiogenic abilities, and inflammatory reactions of cobalt-based biomaterials are caused by the different amounts of released cobalt. In addition, it has been reported that the biological effects (including osteoimmunomodulation, angiogenic abilities, osteogenic activities and angiogenic reaction) of other bioactive ions (Si, Sr, Zn, and Mg) is closely related to their concentration 26-30. These data indicate that tuning the dose range of cobalt ions may be a valid strategy to resolve the controversies about cobalt use for orthopedic applications.

After sophisticated research into bone biology, it is now recognized that the bone regeneration outcome is the result of the cooperation of osteoimmune, skeletal, and vascular systems 31-33. Accumulating evidence indicates that the immune system regulates the balance of bone formation and bone resorption through the production and release of a large spectrum of interacting regulatory molecules 33-35. Besides, it has been demonstrated that an immune response participates in the process of neovascularization by inducing production and release of proangiogenic factors 36. Hence, the ultimate bone-regenerative outcome of biomaterials is determined by these interacting components.

The cobalt-induced bone-regenerative effect caused by one single system has been elucidated in other studies 24, 31, 37. These data have resulted in a better understanding of the function of one of these systems or a specific pathway of cobalt-induced biological activities 38. Nevertheless, inconsistent conclusions can be made from different studies 20, 21, 24. These contradictory results about the biological effects of cobalt may be due to the focus on the effect on one of the systems, which may lead to the negligence of the interaction with others. In addition, a new concept, namely systems immunology, has been proposed; it suggests that an immune response is a complex process involving multiple organs, cell types, cytokines, and pathways 34. Therefore, we can regard, as logical evolution, the process where the research on biomaterial-induced bone regeneration has shifted from focusing on the study of its parts to a broader and more integrated view of how those parts work together to produce particular results. Thus, to prevent the paradoxical effect of cobalt through tuning of its concentration, a deeper understanding of its dose-dependent effect on the cooperation of osteoimmune, skeletal, and vascular systems is needed.

## Materials and methods

### Cell cultures

RAW 264.7 cells (hereafter: RAW cells, a macrophage cell line) were cultured in Dulbecco's Modified Eagle's Medium (DMEM; *Thermo Scientific*, USA) supplemented with 5% of heat-inactivated fetal bovine serum (FBS; Thermo Scientific) and 1% (v/v) penicillin/streptomycin solution (Thermo Scientific), hereafter referred to as the complete medium, in a humidified atmosphere containing 5% of CO_2_, and the temperature set to 37 ℃. The cells were gently scraped off and passaged when they reached ~90% confluence and were expanded *via* two passages before the use in the following experiments.

Bone marrow stromal cells (BMSCs) were isolated and cultured as previously described 39. Briefly, bone marrow was collected from the femurs and tibias of 4-week-old male Sprague-Dawley rats. The isolated cells were transferred to culture flasks containing the culture medium (DMEM supplemented with 10% of FBS and 1% [v/v] penicillin/streptomycin) and incubated in a humidified incubator (37 ℃, 5% CO_2_). Unattached hematopoietic cells were removed *via* culture medium changes, and the attached cells were passaged using trypsin when they reached 90% confluence. Passages 3 to 5 of BMSCs were used in this study 40. Blood was collected from the rats for isolation of peripheral blood mononuclear cells (PBMCs).

The latter were isolated by Ficoll-Hypaque density gradient centrifugation as previously described 41. Briefly, peripheral blood was collected into ethylenediaminetetraacetic acid (EDTA) anticoagulant tubes and diluted with phosphate-buffered saline (PBS; Sigma-Aldrich, Germany) at a ratio of 1:1 before layering onto Histopaque 1077 (Sigma-Aldrich, Germany) in 15 ml centrifuge tubes. The PBMCs were isolated following the instructions of the manufacturer. After erythrolysis with red blood cell lysis buffer (Beyotime Biotechnology, China), the isolated cells were washed with PBS two to three times. The PBMCs were resuspended in the RPMI 1640 medium (GIBCO; Invitrogen, USA) supplemented with 10% of FBS and 1% penicillin/streptomycin and incubated in a humidified incubator (37 ℃, 5% CO_2_).

### Cell viability at various cobalt ion concentrations

A Cell Counting Kit-8 (CCK-8; Dojindo, Japan) assay was used to evaluate the cell viability of RAW cells and BMSCs at different concentrations of Co^2+^ in the complete medium (0, 0.1, 0.5, 1, 5, 10, 50, and 100 ppm), which were prepared with CoCl_2_. RAW cells and BMSCs were seeded at a density of 2,000 cells per well (in a 96-well plate) and cultured overnight. The culture medium was next removed and replaced by a medium containing CoCl_2_. On day 1, 2, 3, 5, 7, 9 the medium was removed followed by addition of a 10% CCK-8 solution. After 2-h incubation, the absorbance of each well was measured on a microplate reader at a wavelength of 450 nm. For cytoskeletal staining, BMSCs and RAWs were seeded into 24-well plate at a density of 10^4^ per well. The stimulation of CoCl_2_ was performed in the same approach as CCK-8 assay. Fluorescence microscopy was performed at 1, 2, 3, 5, 7, 9 days. BMSCs and RAWs cells were fixed by 4% paraformaldehyde for 10 min. After being washed by PBS, the fixed cells were permeabilized using 0.1% Triton/PBS for 5 min. To stain the cytoskeleton, Alexa Fluor 594 phalloidin (1:40 dilutions) were added and incubated at 4 ℃ overnights. Then 2-(4-Amidinophenyl)-6-indolecarbamidine dihydrochloride (DAPI) was added to stain the nuclei for 5 min at room temperature in the dark. Images were captured by microscope (Axio Observer Z1M, Zeiss, Germany).

### Flow cytometry for detection of apoptosis and necrosis

After 2 days, the PBMCs cultured with different concentrations of Co^2+^ (0, 0.1, 0.5, 1, or 5 ppm) were collected for the detection of apoptosis and necrosis. After a wash with PBS, the cells were resuspended in 400 μl of Annexin V binding buffer (BestBio, China) and adjusted to 10^6^ cells/ml. The cells were fluorescently labeled by the addition of 5 μl of Annexin V‐FITC (BestBio) and were incubated at 4 ℃ for 15 min. Subsequently, 10 μl of propidium iodide (PI) solution (BestBio) was added and incubated for 5 min before analysis by flow cytometry (Cytoflex, Beckman Coulter, USA).

### Animal surgical procedure

Sprague-Dawley rats at 8-10 weeks of age were subjected to *in vivo* experiments to evaluate the dose-dependent effects of cobalt *in vivo*. Animal surgical protocols were approved by the Institutional Animal Care and Use Committee (IACUC) of Sun Yat-sen University. The rats were under general isoflurane anesthesia during the surgical procedure. Blood clots containing different doses of Co^2+^ were prepared in a manner similar to the method described in a previous study 42. Rat blood was collected from the tail vein and mixed with a CoCl_2_ solution (1, 5, 10, or 50 ppm) in a 9:1 (v/v) ratio to attain at the final concentration of 0.1, 0.5, 1, or 5 ppm cobalt. Each blood clot was made into 200 μl. After 15-20-min incubation the blood clots were formed. Two circular bone defects of 5-mm diameter were separately created on the left and right side of the rat calvarium using a drill. The blood clots were implanted into the defects properly, leading the dose of the administrated cobalt for each group was 0 ng (0 ppm group), 40 ng (0.1 ppm group), 200 ng (0.5 ppm group), 400 ng (1 ppm group) and 2000 ng (5 ppm group) respectively. The incisions were then closed. The animals that received different concentrations of cobalt were euthanized after 2 days or after 6 weeks.

### Systemic toxicity of cobalt implantation *in vivo*

To evaluate the systemic toxicity, 2 days after the implantation of cobalt, the animals (Each group contained 3 animals) were euthanized, and blood was collected through cardiac puncture. Plasma was separated to measure the cobalt concentration by inductively coupled plasma mass spectrometry (Thermo Scientific). The relevant organs and tissues (the liver, spleen, thymus, cervical lymph node, brain, kidney and heart) were collected for histological staining. For H&E staining, the cell nuclei were stained with Mayer's hematoxylin (Sigma-Aldrich), followed by cytoplasm and extracellular-matrix staining with eosin (Sigma-Aldrich). The brain tissues were also collected for Nissl's staining with cresyl violet (Solarbio, China). The blood clots remaining in the calvarium defects were collected for the experiment described in next section.

### The reaction of the osteoimmune, skeletal, and vascular systems to cobalt implantation *in vivo*

The blood clots remaining in the calvarium defects were collected as described above. The total RNA from the blood clot samples was extracted with the TRIzol reagent (Beyotime Biotechnology, China). Relative mRNA expression levels of immune-system-related factors, bone remodeling-related factors, fibrogenesis-related factors, and angiogenesis-related factors were analyzed by reverse-transcription quantitative PCR (RT-qPCR). RT-qPCR primers used in this study are listed in Supplementary Table 1. SYBR Premix Ex Taq™ (Takara, Japan) was employed for assays on a Light Cycler Real Time PCR machine (Roche, Germany). The average values of three independent tests for each gene were normalized by Z-score after being taken the logarithm of 2. Then clustering heatmap was made by MeV software (Multi Experiment Viewer, version 4.9.0, http://www.tm4.org/mev.html). The interaction of these factors was analyzed on the STRING website (*https://string-db.org/cgi/input.pl?sessionId=Cuzp3bpg46U5&input_page_active_form=multiple_identifiers*).

### The osteogenic effects of cobalt *in vivo*

**Micro-computed tomography (CT) analysis.** The animals (each group contained 3 animals) were euthanized 6 weeks after the surgical procedure to evaluate the new bone formation. The calvarium tissues were collected and fixed with 4% paraformaldehyde before a scan in a Micro-CT scanner (μCT50; SCANCO Medical AG, Switzerland) at a resolution of 15 µm, a source voltage of 70 kV, and a current of 114 µA. The three-dimensional images were reconstructed and the BV/TV were calculated in analysis software Materialise Mimics Research 19 (Materialise, Belgium).

**Staining of histological sections.** The calvarium samples were decalcified in 4% EDTA for 4 weeks. After embedding in paraffin, the samples were sectioned into 4-µm slices for H&E staining as described above. To further assess the expression of osteogenic factors and the formation of blood vessels, immunohistochemical (IHC) staining of alkaline phosphatase (ALP) and α-smooth muscle actin (α-SMA) was applied. Endogenous peroxidase activity was eliminated by incubation in 3% H_2_O_2_ for 15 min. The slides were then blocked for 1 h and incubated with a rabbit monoclonal antibody against ALP (1:200; Abcam, USA) and a rabbit polyclonal antibody against α-SMA (1:200; Abcam) overnight at 4 ℃. The sections were then incubated with a goat anti-mouse IgG or anti-rabbit IgG antibody (Gene Tech, China) (secondary antibody) for 30 min at room temperature. The antibody-antigen complexes were visualized with a diaminobenzidine (DAB) solution (Gene Tech, China) and counterstained with Mayer's hematoxylin for 2-3 min. To confirm the differentiation of osteoclasts, TRAP staining was performed. Samples were incubated in 0.2 M sodium acetate dissolved in distilled water (Sigma-Aldrich) and 0.05 M sodium L-tartrate dihydrate (Sigma-Aldrich) for 20 min at room temperature. Then, naphthol AS-MX phosphate powder (Sangon Biotech, China) and fast red TR salt powder (Aladdin, USA) were added and mixed to prepare TRAP buffer. The samples were then incubated in TRAP buffer for 1-2 h. After a rinse in distilled water, the samples were counterstained with Mayer's hematoxylin (Sigma-Aldrich). Histological images were scanned and captured on the Aperio AT2 system. The relative quantitative analysis was performed by Aperio Image Scope (Leica Biosystem Imaging, Inc.).

### Statistical analysis

All procedures and analyses were performed blinded to animal or cell identity. All data are presented as mean ± standard deviation (SD). To determine if differences existed within groups, data was analyzed by a one-way ANOVA, as appropriate. If such differences existed, Tukey's multiple comparisons test was used to determine the group(s) with the difference(s) (Prism 8.00; GraphPad Software, Inc.). A final value of P<0.05 was considered significant for all analyses.

## Results

### Cytotoxicity of cobalt

**Figure 1:**
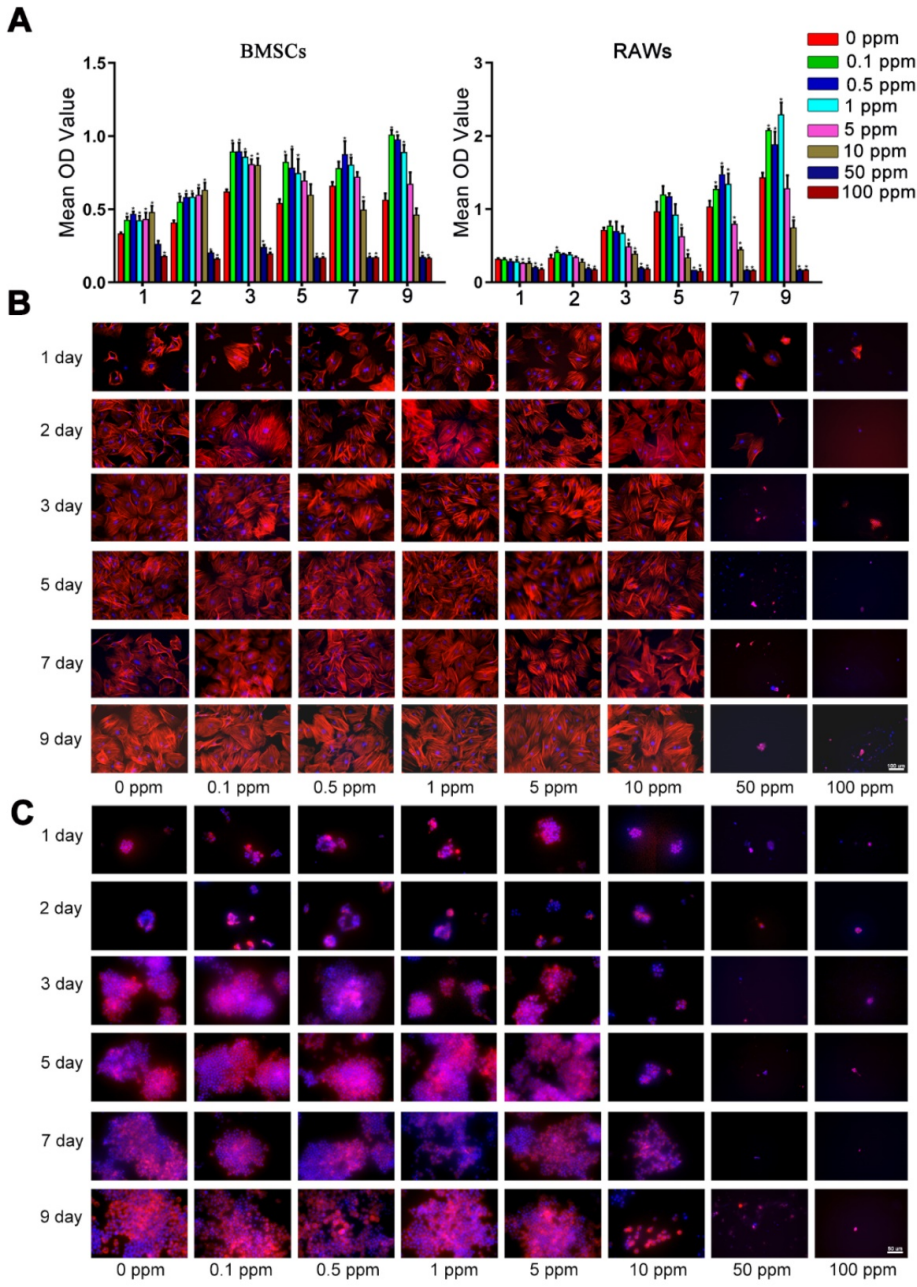
Cellular toxicity of cobalt. A. The viability of BMSCs and macrophages in the media containing different concentrations of CoCl_2_ (0, 0.1, 0.5, 1, 5, 10, 50, or 100 ppm), as determined by the CCK-8 assay (*0 ppm group vs 0.1-5 ppm groups, p < 0.05). B, C. Fluorescence images of cytoskeletal staining shows the cell density and morphology of BMSCs (B) and RAWs (C).

The high cobalt concentration (50-100 ppm) exerted inhibitory effects on the proliferation of BMSCs, because the OD value of the CCK-8 assay decreased dramatically in the 50 and 100 ppm groups after 2-day cultivation (Figure 1A). In the 10 ppm group, a significant decrease was observed at 7 day. The toxic effect of cobalt on the macrophages was stronger. In the 5 ppm group the OD value slightly decreased at 1, 3, 5, 7 day but no significant change was observed on day 9. The OD value of the 10 ppm group decreased at 1, 3, 5, 7 and 9 day. A significant decline of cell viability was observed at all the experimental time points in the 50 and 100 ppm groups (Figure 1 B). Consistent with the results of CCK-8, the cytoskeletal staining showed that in 50 and 100 ppm, the number of BMSCs remarkably dropped and the abnormal morphology obviously appeared, such as pyknosis, karyorrhexis. As for macrophages, the cell number markedly decrease in10, 50 and 100 ppm groups. The cells were almost could not be detected in high cobalt concentration groups (50 and 100 ppm). To confirm the safe concentration of cobalt, the apoptosis of PBMCs was tested after treatment with 0-5 ppm cobalt for 2 days. The results of the Annexin V/PI flow cytometry showed high cell viability (97-98% cells tested negative for Annexin V and PI staining) in all these groups (Supplementary Figure 1).

### Systemic toxicity of cobalt *in vivo*

**Figure 2:**
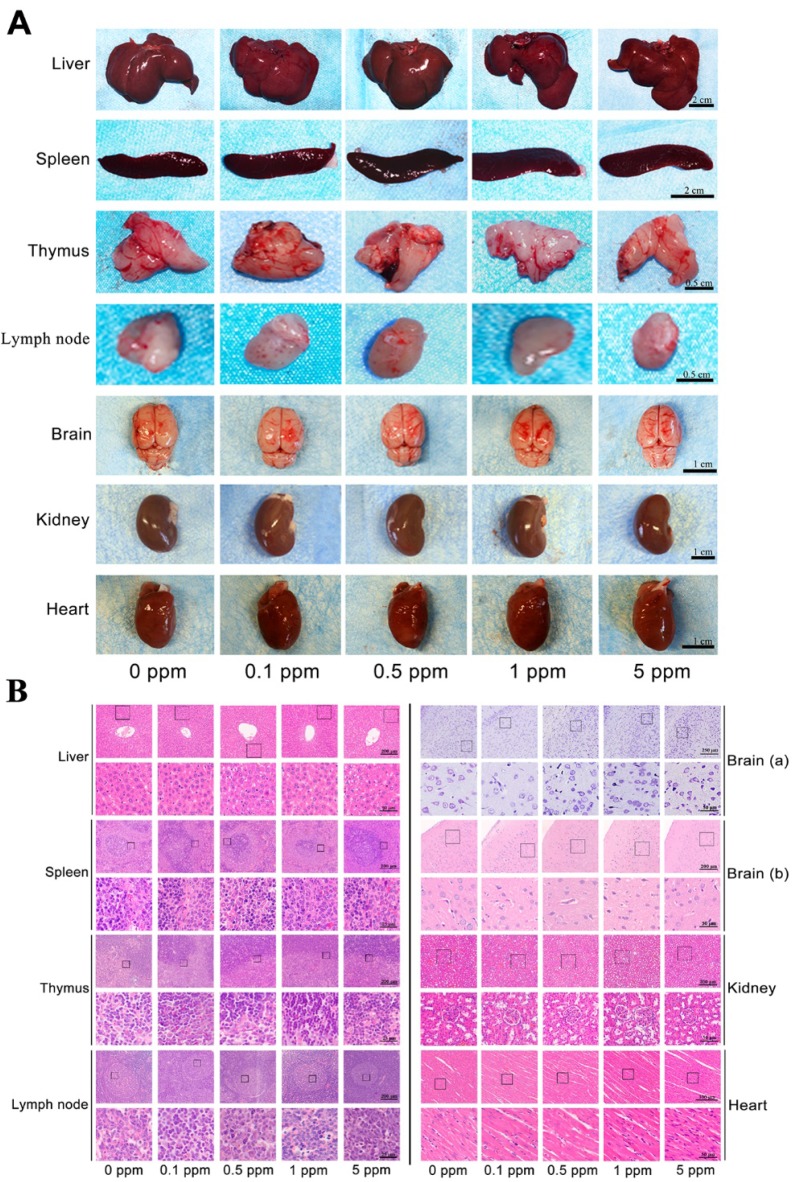
*In vivo* rapid systemic toxicity of cobalt. A. Macroscopic examination of the liver, spleen, thymus, lymph node, brain, kidney and heart in the 0-5 ppm cobalt groups. B. Microscopic view of the liver, spleen, thymus, lymph node, brain, kidney and heart in the 0-5 ppm cobalt groups. H&E staining shows that the tissue structure in all the organs. Nissl's staining shows the structure of neurones. No toxic effect was observed.

Before investigating the *in vivo* osteogenic ability of cobalt, further confirmation of the safety of cobalt at the applied concentrations was needed. Cobalt at different concentrations (0 to 5 ppm) was applied to the rat model of calvarial bone defects. The cobalt concentration in plasma 2 days after implantation ranged from 0.016 to 0.054 ppm among different groups (Table 1). These concentrations were well below the* in vivo* cobalt toxicity level, which has been reported to be 0.3 ppm in another study 6. From the view of macroscopic examination, *H&E staining and Nissl's staining*, among all the groups, no toxic reaction including hyperemia, ischemia, atrophy, or necrosis was observed in the liver, spleen, thymus, and lymph node, brain, kidney and heart (Figure 2). Thus, cobalt at 0 to 5 ppm did not manifest rapid systemic toxicity and could be applied in further* in vivo* experiments.

### The reaction of the osteoimmune, skeletal, and vascular systems to cobalt at an early stage

The biomaterial-induced bone regeneration is jointly regulated by osteoimmune, skeletal, vascular systems, which involve a large spectrum of interacting regulatory factors. For this reason, the expression profile of these factors at different cobalt concentrations should be considered during the development of cobalt-based bone-regenerative materials. According to previous studies, these factors include tumor necrosis factor α (TNFα), interferon γ (IFNγ), interleukin 1β (IL1-β), IL-6, IL-10, interleukin 1 receptor antagonist (IL-1Ra), transforming growth factor β 1 (TGFβ1), osteoprotegerin (OPG), receptor activator of nuclear factor-κ B ligand (RANKL), receptor activator of nuclear factor-κ B (RANK), TNF receptor-associated factor 6 (TRAF6), nuclear factor light chain enhancer of activated B cells (NF-κB), inhibitor of NF-κB (IκB), c-Jun N-terminal kinase (JNK), oncostatin M (OSM), signal transducer and activator of transcription 3 (STAT3), osteopontin (OPN), osteocalcin (OCN), bone sialoprotein (BSP), alkaline phosphatase (ALP), runt-related transcription factor 2 (RUNX2), bone morphogenetic protein 2 (BMP2), BMP6, collagen type I α 1 chain (COLA1), Wnt5a, Wnt3a, Wnt10b, Axin2, β-catenin, vascular endothelial growth factor A (VEGFA), platelet-derived growth factor subunit a (PDGFa), platelet-derived growth factor subunit b (PDGFb), matrix metalloproteinase 9 (MMP9), CD31, endothelial nitric oxide synthase (eNOS), angiopoietin 1 (Ang-1), and α-SMA.

**Figure 3:**
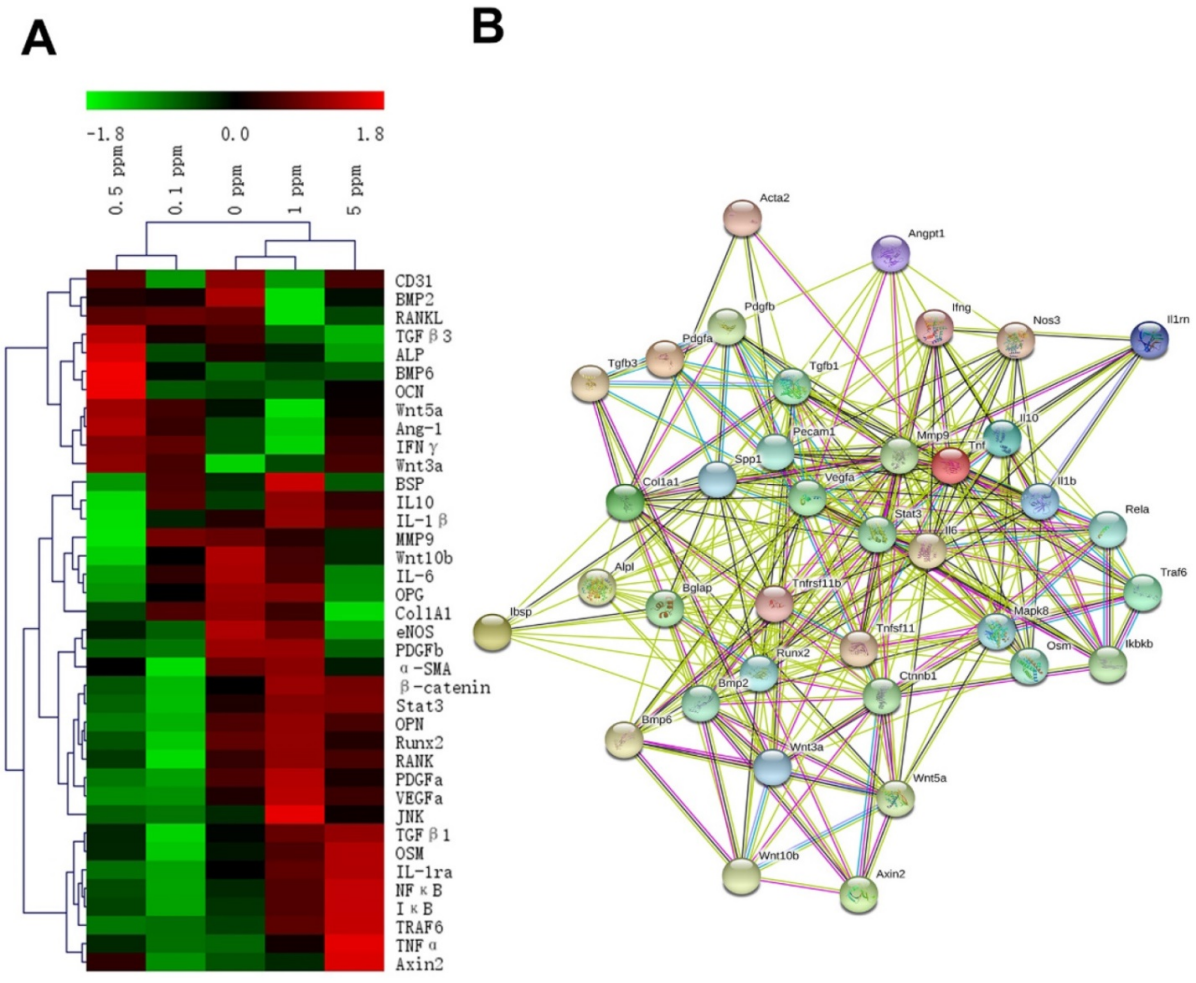
The reaction of the osteoimmune, skeletal, and vascular systems to cobalt at the early stage. A. The clustering heatmap depicts the relative mRNA expression of the multisystem cytokines. Red color indicates the genes whose expression increased, and the green color denotes the genes whose expression decreased. B. The interaction network of the multisystem cytokines.

These factors are related to the processes of an immune reaction, osteogenesis, fibrillogenesis, and angiogenesis, and their expression patterns were revealed by the RT-qPCR analysis at 2 days after the surgical procedure. The gene expression heatmap showed the cytokine profiles of different groups (Figure 3A). The groups with different concentrations of cobalt manifested different expression patterns of these factors, thereby indicating a dose-dependent effect of cobalt on osteoimmune regulation. The interaction network of these factors suggested that these factors were closely related to one another (Figure 3B).

### The influence of cobalt on bone regeneration *in vivo*

**Figure 4:**
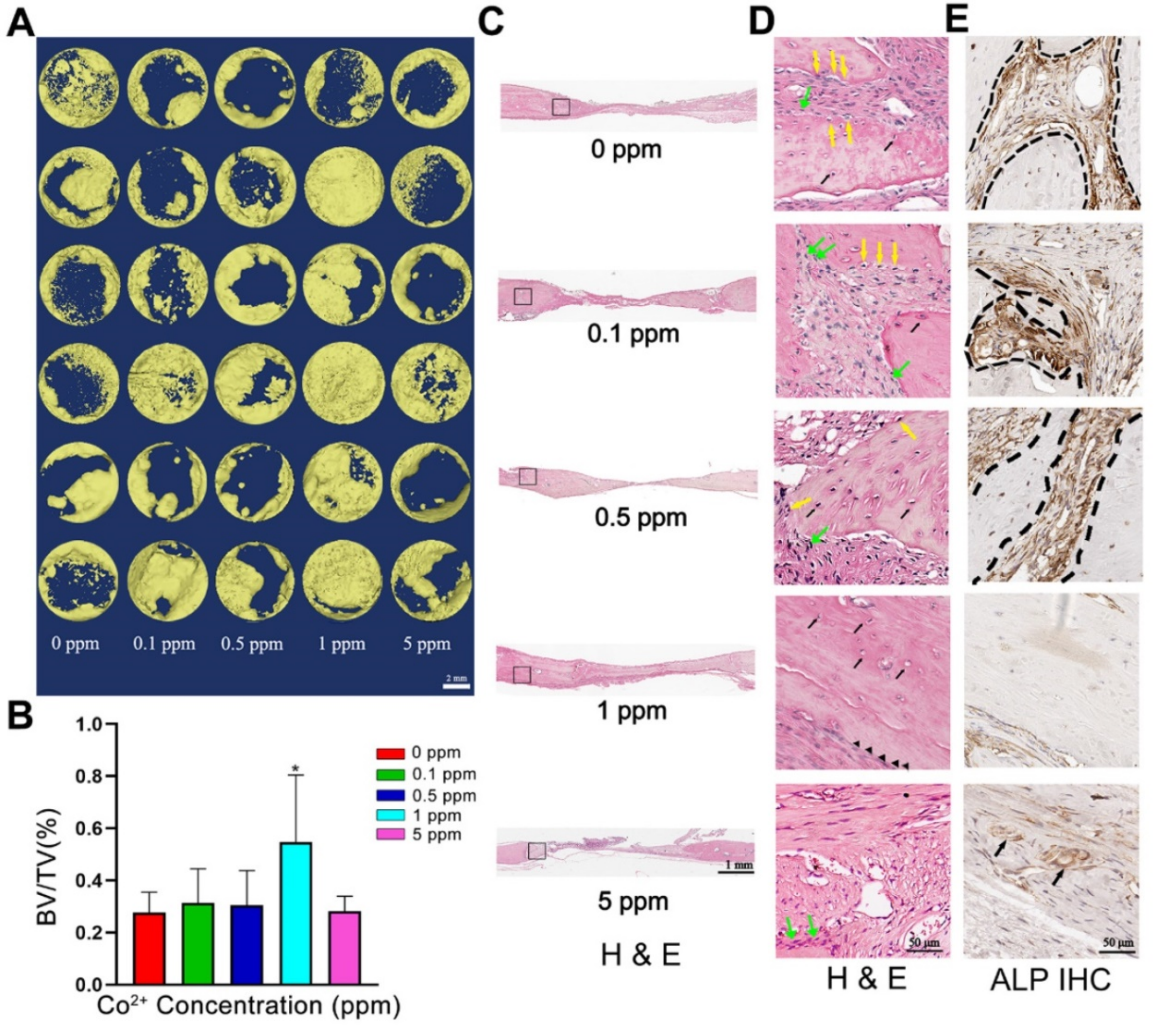
The *in vivo* bone-regenerative effect of cobalt. A. Three-dimensional reconstruction images of the newly formed bone in the 0-5 ppm groups. B. Newly formed Bone volume ratio in the 0-5 ppm groups (*0 ppm group vs 0.1-5 ppm groups, p < 0.05). C. An overview of the calvarium defect area (H&E staining). D. Higher magnification of brackets in panel. Mature bone lamella (black triangle) were observed in the 1 ppm group, and osteocytes (black arrow) were found in the bone lacuna. Immature woven bone formed in the 0, 0.1, and 0.5 ppm groups. Multiple cell types including osteoprogenitor cells (green arrow), osteoblasts (yellow arrow), and osteocytes (black arrow) were noted. In the 5 ppm group, the defect area was mainly occupied by fibrous tissue. E. ALP IHC staining. A large number of ALP-positive cells was found in the 0, 0.1, and 0.5 ppm groups. Limited numbers of ALP-positive cells were observed in the 1 ppm group. BV, bone volume. TV, total tissue volume.

To investigate the osteogenic effect of cobalt *in vivo*, the calvarial bones of the experimental rats were collected 6 weeks after the surgical procedure. As depicted in the micro-CT image, the calvarial-bone defect was repaired most effectively in the 1 ppm group, judging by the largest regenerative bone area and the thickest newly formed bone. The 5 ppm cobalt group manifested the worst bone-regenerative effect (Figure 4A, B).

**Figure 5:**
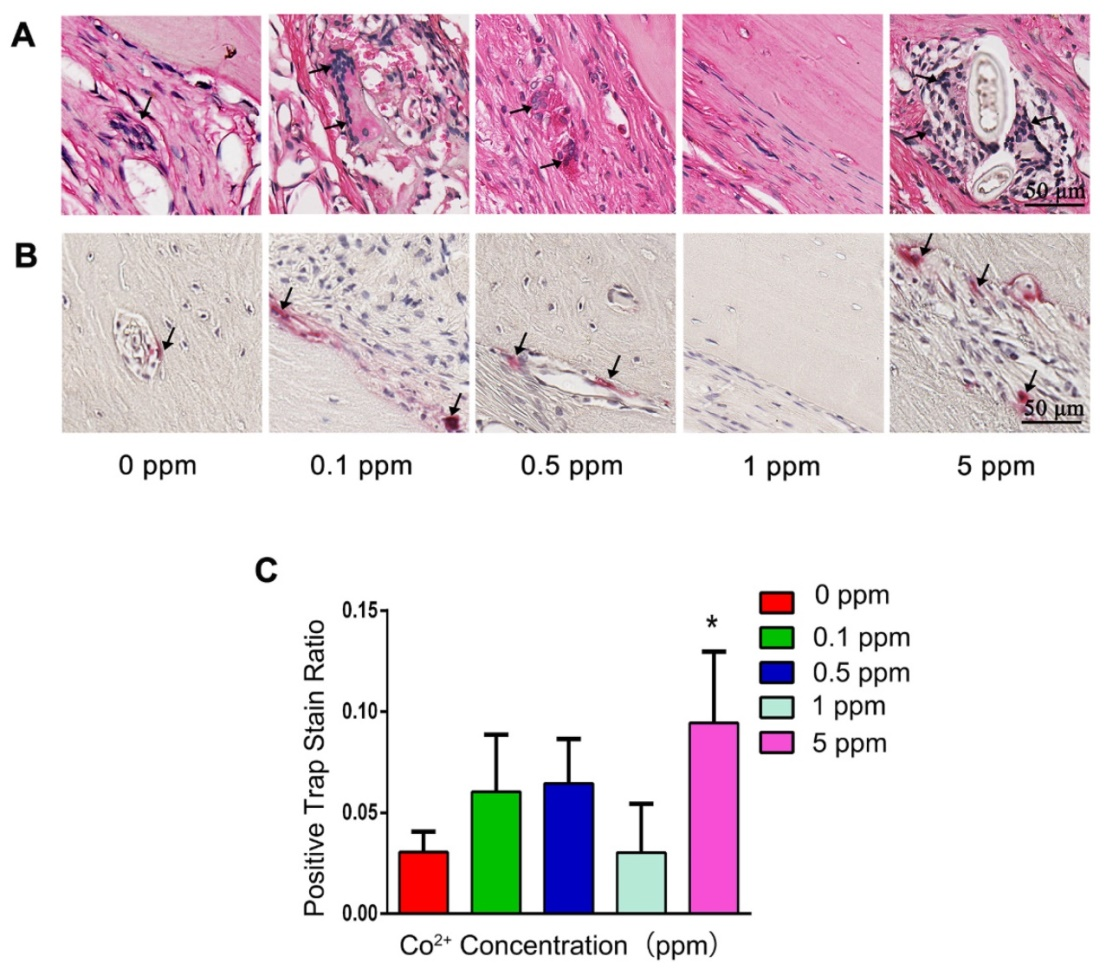
The impact of cobalt on osteoclastogenesis. A. H&E staining shows the formation of osteoclasts (black arrow). B. TRAP staining for the confirmation of detection of osteoclasts (black arrow). C. Relative quantification of the TRAP-positive cells (*0 ppm group vs 0.1-5 ppm groups, p < 0.05).

In the 0, 0.1, and 0.5 ppm groups, nascent bone tissue was observed at the defect ends. A few bone islands appeared in the defect area and were wrapped by collagen fibers (Figure 4C). The newly formed bone tissue was immature woven bone with disorderly arranged collagen fibers and insufficient mineralization. Various cell types were observed in these groups, thus indicating an active bone-regenerative process. The osteoprogenitor cells were found in the fibrous tissue, and the osteoblasts covered the newly formed bone surface (Figure 4D). The presence of osteoblasts was then confirmed by ALP IHC staining. A large number of ALP-positive cells were observed on the boundary between the newly formed bone and the expanding fibrous tissue (Figure 4E). In addition, the osteoclasts in the 0.1 and 0.5 cobalt ppm groups were suggestive of active bone remodeling in these groups (Figure 5).

Newly formed bone was observed in the whole defect area in the 1 ppm group, and the mineralized bone tissue connected the defect ends (Figure 4C). At high magnification, mature lamellar bone was observed in the 1 ppm group. The collagen fibers in the mineralized bone tissue were well organized and parallel to the bone surface (Figure 4D). The ALP-positive cells and osteoclasts were seldom observed, suggesting that the bone regeneration process was at the late stage (Figures 4E and 5).

In the 5 ppm group, the bone regeneration process was at a very early stage and was dominated by fibrous-tissue expansion. Only little bonelike tissue was observed on the defect boundary, and no newly formed bone tissue was detected in the defect area. A thin layer of collagen connected the defect ends (Figure 4C). The collagen fibers grew slightly and were arranged randomly. The extracellular matrix was not mineralized (Figure 4D). Significantly increased numbers of osteoclasts were observed in the 5 ppm groups (Figure 5). Therefore, 1 ppm cobalt could significantly enhance the bone regeneration *in vivo*, while the 5 ppm cobalt hampered the bone-healing process.

### The influence of cobalt on vascularization *in vivo*

**Figure 6:**
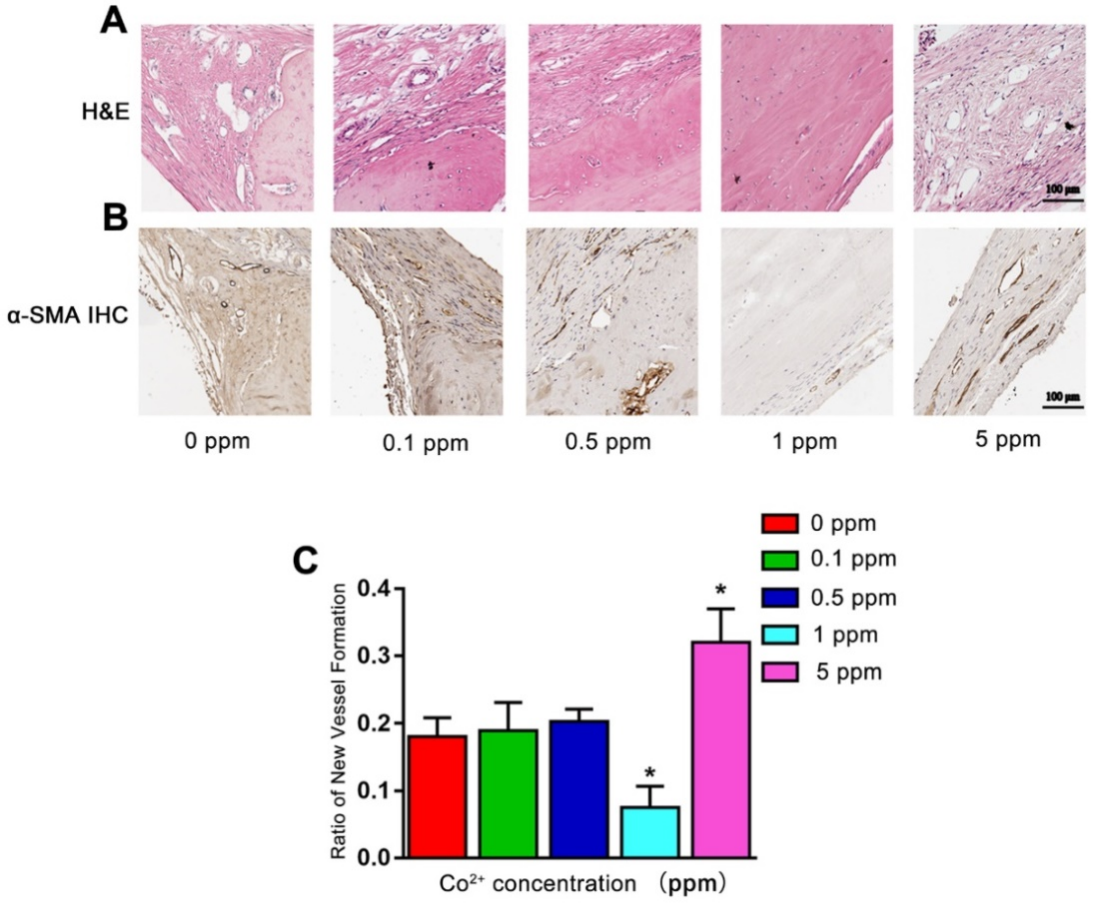
The angiogenic effect of cobalt in 6 weeks. A. H&E staining shows that 6 weeks after the surgical procedure, in the 0, 0.1, and 0.5 ppm groups, plenty of newly formed blood vessels were noted. In the 1 ppm group, only a few blood vessels could be found on the boundary of bone and collagen. In the 5 ppm group, numerous blood vessels were found in the fibrous tissue. B. The formation of blood vessels was confirmed by α-SMA IHC staining. C. Relative quantification of the α-SMA-positive cells (*0 ppm group vs 0.1-5 ppm groups, p < 0.05).

Angiogenesis also plays an important role in bone defect repair. At 6 weeks after the surgical procedure, in the 0, 0.1, and 0.5 ppm groups, plenty of newly formed blood vessels were found in the area where the active remodeling of bone occurred. In the 1 ppm group, the bone-regenerative process was at the mature stage with only a limited number of blood vessels could be found. In the 5 ppm group, numerous blood vessels were seen in the fibrous tissue, implying the formation of granulation tissue (Figure 6A). The formation of blood vessels was confirmed by α-SMA IHC staining (Figure 6B). The relative quantity of α-SMA-positive staining is shown as Figure 6C. These results collectively suggested that among different cobalt concentration groups, 1 ppm cobalt accelerated the bone-regenerative process most strongly.

### The mechanism underlying the cobalt-induced osteogenic regeneration

**Figure 7:**
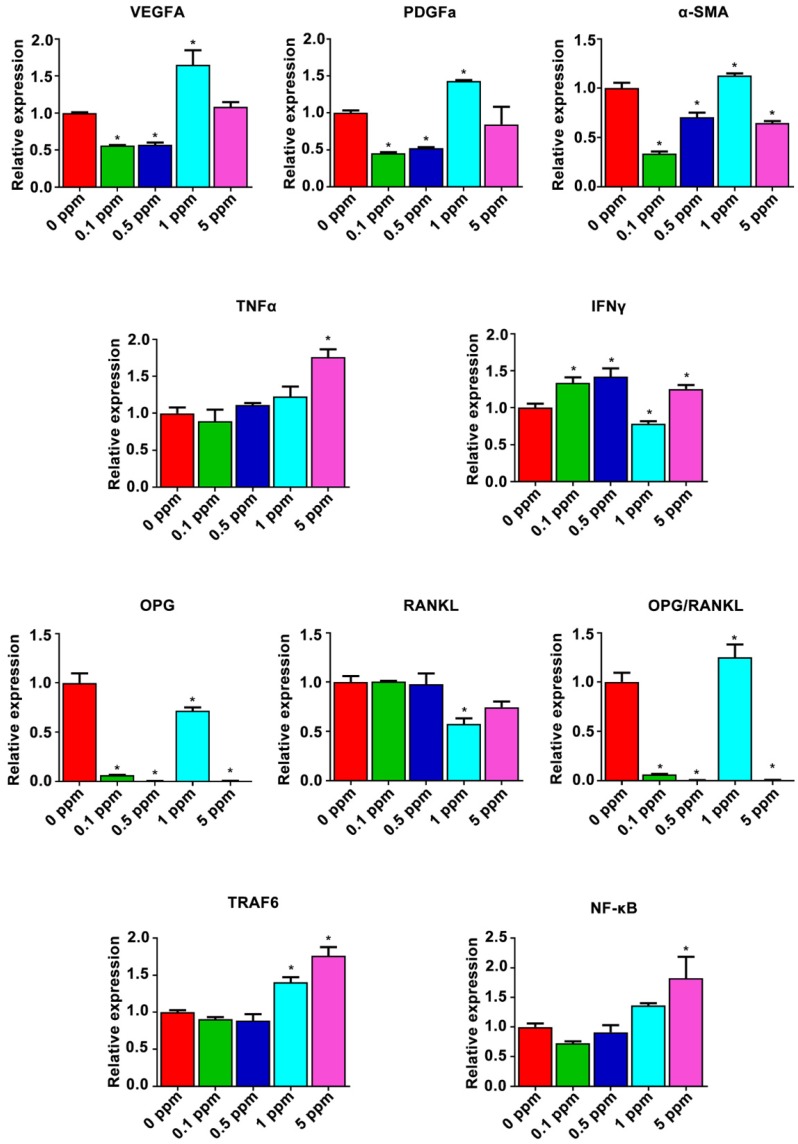
The possible mechanism underlying the cobalt-induced bone regeneration. The relative mRNA expression of genes from the osteoimmune, skeletal, and vascular systems including VEGFA, PDGFa, α-SMA, TNFα, IFNγ, OPG, RANKL, TRAF6, and subunits of NF-κB.

Possible mechanism underlying the dose effect of cobalt on bone regeneration was analyzed form the results of the gene heat map. The relative expression of proangiogenic factors VEGFA, PDGFa, and α-SMA and of osteoimmune factors TNFα, IFNγ, OPG, RANKL, TRAF6, and subunits of NF-κB was further analyzed (Figure 7). The relative expression patterns of VEGFA and PDGFa were similar, increasing in the 1 ppm group and slightly decreasing in the 0.1 and 0.5 ppm groups at the early stage. By contrast, the relative expression of α-SMA increased in the 1 ppm group and decreased in the 0.1, 0.5, and 5 ppm groups, indicating an active angiogenic reaction in the 1 ppm group at the early stage. There was a significant increase in the mRNA expression of TNFα in the 5 ppm group (1.8-fold), whereas no remarkable change was observed in the other groups. The expression of IFNγ increased in the 0.1, 0.5, and 5 ppm groups, but decreased in the 1 ppm cobalt group. The comparison of OPG and RANKL expression levels revealed a relatively higher OPG/RANKL ratio in the 1 ppm group compared to the other groups. TRAF6 and NF-κB are the downstream factors of the RANKL/RANK pathway. TRAF6 can also be degraded under the influence of IFNγ. The expression of TRAF6 turned out to be upregulated in the 1 and 5 ppm groups, and the expression of subunits of NF-κB was high in the 5 ppm group.

## Discussion

In our study, we tried to resolve the controversies about cobalt use in orthopedic applications, through the strategy of tuning the cobalt dose range to manipulate the cooperation of osteoimmune, skeletal, and vascular systems. It was found that cobalt at high doses (>10 ppm) had significant negative effects on the growth of macrophages and BMSCs. Lower concentrations (0.1-5 ppm) of cobalt did not have toxic effects on BMSCs, macrophages, and PBMCs. No systemic toxicity *in vivo* was noted among low-concentration groups (0.1-5 ppm). The cytokine profiles of the multiple interacting systems were different in different dose groups, and these cytokines are engaged in broad interactions. For instance, 1 ppm cobalt had the optimal comprehensive effect on systematic osteoimmunomodulation, early angiogenesis, and bone tissue regeneration.

### The dose-dependent toxic effects of cobalt

Toxicity is a major concern for the application of cobalt-based biomaterials according to the traditional paradigm, which regards cobalt as a toxic agent 42, 43. Although this stereotype has changed with a more sophisticated understanding of the biological function of cobalt, higher concentrations of cobalt may cause an excessive inflammatory reaction, endocrine disorder, and adverse effects on development 43. Therefore, to determine a safe range for cobalt-based biomaterials and protect the researchers as well as the experimental animals, the toxicity of the cobalt was evaluated first.

According to other studies, macrophages 44 and PBMCs 33, 45 are the major immune cells mediating biomaterial-induced osteoimmunomodulation. BMSCs are regarded as the target cells in bone regeneration 44, 46. Although the CCK-8 results indicated that 5 ppm cobalt would affect the proliferation of macrophages on day 3, 5 and 7, from the view of cytoskeletal staining no toxic reaction was observed because the cell amount and morphology of the 5 ppm groups were similar to the control groups. In addition, no significant decreased was observed in the 5 ppm group on day 9 in CCK-8 assay. Based on these results, 5 ppm is approximate to the toxic margin of cobalt on macrophages. It would be difficult to judge that whether the 5 ppm concentration of cobalt has a toxic effect in *in vivo* application, thus its systemic toxicity and the comprehensive biological effect were further investigated in this study. This result is similar to the findings of another study, which showed that 6 ppm is the toxic level of cobalt for mouse macrophages 47. Our toxic concentration is much lower than the toxic level reported for primary human lymphocytes: 30 ppm 43, 48. The toxic thresholds were different in different cell types; therefore, to ensure the safety of *in vivo* application, the applied cobalt concentration should be set to the lower one, and an *in vivo* toxicity test is necessary.

Besides the local tissue, cobalt-based biomaterials interact with body fluids and multiple organ systems after implantation, because cobalt ions and particles can be generated and released due to tribocorrosion 49-51. Hence, a rigorous standard with consideration for systemic adverse reaction seems more sensible for evaluation of the toxic effects of cobalt. The liver, thymus, lymph node, spleen, brain, kidney and heart play an important role in the interaction with a foreign body (implant) 52-55; hence, they were selected to be the representative organs for examination of the systemic toxicity of cobalt. In this study, the plasma cobalt concentrations were much lower than the detrimental concentration reported elsewhere, which was 0.3 ppm 6. No adverse reaction and damage were observed in the tested organs. Therefore, 0.1-5 ppm cobalt did not manifest rapid toxicity after implantation and could be tested in subsequent experiments.

### The dose-dependent systematic (osteoimmune, skeletal, and vascular) response *in vivo* after treatment with cobalt

In this study, the *in vivo* samples were collected to detect the osteoimmune cytokine profile, which may reflect the real *in vivo* scenarios more accurately. According to our results, after 1 ppm cobalt exposure, angiogenic factors such as VEGFA and PDGFa were upregulated, indicating active vascularization in the early stage. The formation of vessels could promote the migration of inflammatory cells and the release of inflammatory cytokines, thereby initiating an inflammatory response 56. Anti-inflammatory factors such as IL-1Ra, IL-10, and TGFβ were found to be upregulated, thus preventing excessive inflammation and resulting in a moderate immune response. It should be noted that, in late bone forming phase, the new formed bone tissue become mature lamellar bone. Some of the new vessels become mature vessel lumen to suit the deposition of new bone. With the degeneration of granulation tissue the number of vessels decrease 57. Therefore, although the ratio of the vessel formation decreased in the 1 ppm group at the 6 weeks, it did not mean that the 1 ppm cobalt stimulation negatively affects angiogenesis, on the contrary, it is a manifestation for a rapid bone regenerative process.

As to osteogenesis, OSM released by activated macrophages is one of the cytokines of the IL-6 family and has been demonstrated to enhance osteoblastic differentiation through the transcription factor STAT3 with upregulation of BSP 58. OSM, STAT3, and BSP were found to be upregulated in the 1 ppm group, suggesting that the osteogenic environment was beneficial in this treatment group. In addition, IFNγ was underexpressed; it is known as a proinflammatory factor that inhibits BMSC osteogenic differentiation by downregulating the RUNX2 pathway 59, 60.

As to the osteoclastogenesis system, bone formation is determined by the balance of osteogenesis and osteoclastogenesis. The appearance of osteoclast is vital for bone maturation and functioning, however, over activation of osteoclastogenesis may cause negative effect to the bone regeneration.

RANKL is one of the important factors that couples the immune system and bones 44. RANKL can be produced by activated T cells and promotes differentiation into osteoclasts. TRAF6 and NF-κB are the downstream factors of RANKL, both of which participate in bone resorption 61. The effect of RANKL can be blocked by OPG, a soluble decoy receptor mainly secreted by B lymphocytes 62. Moderate osteoclastogenic activity persisted in the 1 ppm group with upregulation of TRAF6 and of the OPG/RANKL ratio at the same time. Thus, in the 1 ppm group, the multimodal regulation of these cytokines jointly generated the so-called favorable osteoimmune microenvironment, which triggered vascularization at an early stage, a moderate inflammatory response, and maintained the balance between bone formation and bone resorption, thereby resulting in better bone regeneration.

After low-dose cobalt treatment (<0.5 ppm), most of the cytokines were slightly downregulated or their expression remained unchanged in the early stage. For example, the expression levels of proangiogenic factors VEGFA and PDGFa, proinflammatory factors IL-1β and IL-6, pro-osteogenic factors OSM and STAT3, and the ratio of osteoclastic-differentiation-related factors (OPG/RANKL) all decreased. At the late stage (6 weeks), active bone remodeling and vascularization were observed, indicating that mature bone regeneration was in progress although lagging behind the 1 ppm group.

After high-dose cobalt treatment, proinflammatory factors TNFα and IFNγ were significantly upregulated, leading to an excessively inflammatory environment. TNFα causes BMSC apoptosis by activating the Fas-signaling-mediated death pathway 63. Aside from inhibiting osteoblastic differentiation, IFNγ has been found to synergistically enhance the TNFα-induced BMSC apoptosis 45. TRAF6 and subunits of NF-κB were markedly upregulated at the early stage (2 day) and osteoclast formation increased at 6 weeks in the 5 ppm group. These data indicated that osteoclastogenic activity was triggered at the early stage and resulted in excessive bone resorption at the late stage. Along with the increase in osteoclastogenesis, vessel formation and fibrogenesis were induced too. All these findings were suggestive of a disequilibrium of the osteogenesis/osteoclastogenesis system with formation of a fibrous capsule and granulation tissue owing to the inferior osteoimmune microenvironment generated by the high dose of cobalt.

**Figure 8:**
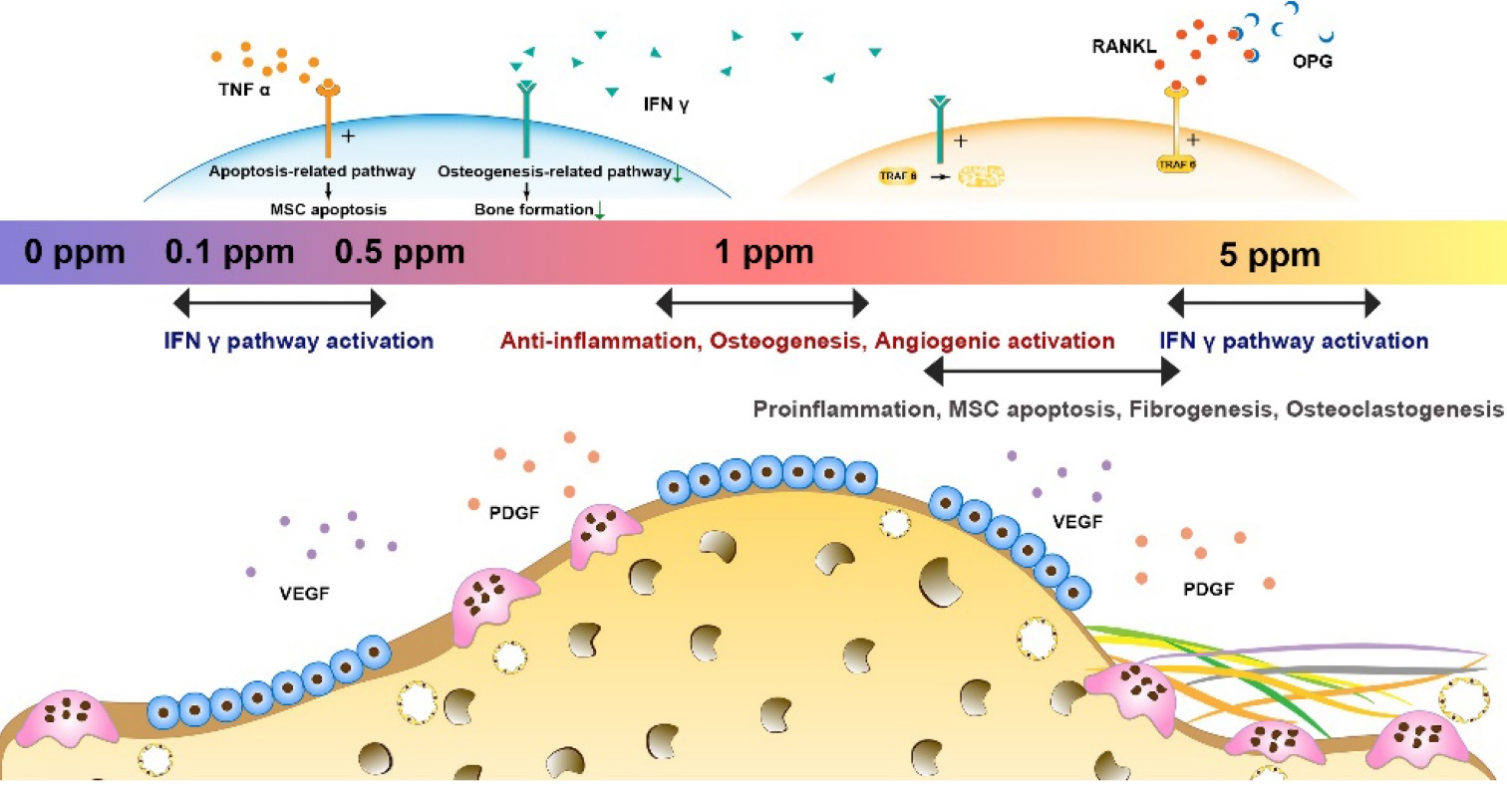
Tuning the cobalt dose range is a promising strategy to resolve the controversies about the orthopedic applications of cobalt. The cobalt-induced bone regeneration is the outcome of the cooperation of osteoimmune, skeletal, and vascular systems. For the multimodal effects of cobalt, appropriate regulation of these multiple systems ultimately yields the optimal bone regeneration effect. In contrast, overactivation of a single system did not always benefit the artificial regeneration effect. An optimal bone regeneration effect was achieved in the 1 ppm cobalt group with a good balance of the osteogenesis/osteoclastogenesis system, a moderate immune response, and superior proangiogenic effects.

In summary, cobalt ions (Co^2+^) exert significant effects on the regulation of the osteoimmune, skeletal, and vascular systems. *Via* tuning of cobalt ion concentration, the osteoimmune environment and subsequent angiogenesis, osteogenesis, and osteoclastogenesis can be manipulated (Figure 8). This notion implies that the strategy of tuning the cobalt dose range can help resolve the controversies about cobalt use in orthopedic applications. The 1 ppm dose of cobalt was superior at promoting the cooperation of these systems and subsequently ensured optimal bone regeneration. In the future, cobalt-containing biomaterials should be developed with the cobalt ion release capacity controlled at ~1 ppm.

### Implications for the development of cobalt-based biomaterials

From the *in vitro* results of our study, cobalt concentration lower than 5 ppm was safe for BMSCs, macrophage and PBMCs, indicating that 5 ppm would not cause local toxicity. *In vivo* evaluation of multi-organs further confirmed that the cobalt concentration below 5 ppm is the safety window.

A biokinetic model for cobalt to characterize the dose-response relationship for cobalt-induced systemic health effects based on a series of human oral dosing studies and toxicology data has been developed. According to the model, when the cobalt levels is below 0.3 ppm, systemic side effect are unlikely to occur. Sever effects including neurologic and cardiac toxicity were only seen at high cobalt level (> 0.7 ppm) 64. The research also suggested to apply a safety factor of 3 to account for inter-individual variability, and it might be useful to start monitoring implant patients from blood cobalt levels of 0.1 ppm. According to our results, the serum cobalt concentration ranged from 0.016±0.020 ppm to 0.026±0.035 ppm in the 0.1 to 5 ppm group. These values were much low than the reported safe level. These collectively indicate the recommended dose range (<5ppm) in this study should be within the safety window of cobalt.

The therapeutic window seemed narrow since the applied concentration in around 1 ppm achieved an optimal vascularized bone regeneration in rat. However, in the bone engineering area, beside the positive effect in vascularization, the superior physicochemical property of cobalt is also a concern. Cobalt-based biomaterials have superior biodegradation-resistance and excellent mechanical properties, achieving certain clinical successes in hip and knee replacements. Therefore, when the applied cobalt in cobalt-based applications, within the scope of safety window but beyond the scope of having an accelerative effect of vascularization, the application of cobalt still has a positive effect on orthopedic application outcome viewing from its improvement of physicochemical property.

Nevertheless, except for cobalt, other bioactive ions such as Si, Cu, Zn, Sr, Mg, etc. 29, 30, 65, 66. also, dose-dependently favor the angiogenic and osteogenic activities. The addition of these bioactive ions may help to improve the angiogenic and osteogenic effect of cobalt. In the development and application of biomaterials, the physical properties, chemical properties and biological effects of the bioactive ions as well as the special effect of the specific one should be comprehensively taken into consideration. Thus, the use of bioactive ions should be rigid and cautious. This may cause limitation for the application of some ions in some situations. More thorough researches for different single ions and the combination of multiple ions could be needed for the development of advanced bioactive ion-contained materials.

## Conclusions

No systemic toxicity was observed *in vivo* at a low dose of cobalt (0.1-5 ppm). The cytokine profiles of the osteoimmune, skeletal, and vascular systems were different in different cobalt dose groups, resulting in different bone regeneration outcomes. The 1 ppm dose of cobalt yielded the most favorable cooperation of the osteoimmune, skeletal, and vascular systems and subsequently optimal bone regeneration outcomes. Tuning the cobalt dose range to manipulate the cooperation of osteoimmune, skeletal, and vascular systems could be a promising and valuable strategy to prevent paradoxical effects of cobalt while preserving its beneficial effects.

## Figures and Tables

**Table 1:** Cobalt concentration (ppm) in plasma of the experimental animal

Animal group (n=3)	0 ppm	0.1 ppm	0.5 ppm	1 ppm	5ppm
Co^2+^concentration (ppm) (Mean ± SD)	0.054±0.057	0.022 ± 0.006	0.016 ±0.020	0.026±0.035	0.022±0.023
