# Supplementary material for: Modulating the cobalt dose range to manipulate multisystem cooperation in bone environment: a strategy to resolve the controversies about cobalt use for orthopedic applications
Source: Theranostics. 2020 Jan 1;10(3):1074–89. doi: 10.7150/thno.37931 (PMC6956813; doi:10.7150/thno.37931)
Supplement: Supplementary file 1 — Supplementary figures and tables. [file thnov10p1074s1.pdf]

Supplementary Table 1. RT-qPCR primers applied in this study

Genes	Primer sequences
**TNFα**	**Forward:** 5'- GGATCTCAAAGACAACCAAC -3'<br>**Reverse:** 5'- ACAGAGCAATGACTCCAAAG -3'
**IFNγ**	**Forward:** 5'- GTGTCATCGAATCGCACCTG -3'<br>**Reverse:** 5'- GTTCACCTCGAACTTGGCGA -3'
**IL1β**	**Forward:** 5'- TCTGTGACTCGTGGGATGAT -3'<br>**Reverse:** 5'- CTTCTTTGGGTATTGTTTGG -3'
**IL6**	**Forward:** 5'- TGATGGATGCTTCCAAACTG -3'<br>**Reverse:** 5'- GAGCATTGGAAGTTGGGGTA -3'
**IL10**	**Forward:** 5'- TCCGGGGTGACAATAACTGC -3'<br>**Reverse:** 5'- TGGCCTTGTAGACACCTTTGT -3'
**IL-1Ra**	**Forward:** 5'- GACCTTCTACCTGAGGAACAACCAG -3'<br>**Reverse:** 5'- AAGAACACATTCCGAAAGTCAATAGG -3'
**TGFβ1**	**Forward:** 5'- GGAAATCAATGGGATCAGTC -3'<br>**Reverse:** 5'- CTGAAGCAGTAGTTGGTATC -3'
**TGFβ3**	**Forward:** 5'- GTTTACCAAGCCAAAGTCC -3'<br>**Reverse:** 5'- GGTGTCTAGCCAAATGTCC -3'
**OPG**	**Forward:** 5'- AAGGAAATGCAACACATGAC -3'<br>**Reverse:** 5'- TCTCTACACTCTCTGCATTC -3'
**RANKL**	**Forward:** 5'- CTCATGCAGGAGAATCAAAC -3'<br>**Reverse:** 5'- TTCCATCATAGCTGGAACTC -3'
**RANK**	**Forward:** 5'- AAAACGCTGACAGCTAATC -3'<br>**Reverse:** 5'- AGTCTTCTGGAACCATCTTC -3'
**TRAF6**	**Forward:** 5'- GCCCATGCCGTATGAAGAGA -3'<br>**Reverse:** 5'- ACTGAATGTGCAGGGGACTG -3'
**NF-κB**	**Forward:** 5'- AACACTGCCGAGCTCAAGAT -3'<br>**Reverse:** 5'- CATCGGCTTGAGAAAAGGAG -3'
**IκB**	**Forward:** 5'- CCTCACCCTTCCCCAATAAT -3'<br>**Reverse:** 5'- GTGTGAATGGTGCCTGTGAC -3'
**JNK**	**Forward:** 5'- CAAGGAGGTCATGGATTTGG -3'<br>**Reverse:** 5'- AAGACGACGGATGCTGAGAG -3'
**OSM**	**Forward:** 5'- GAGAACACTGCTTAGTTTGG -3'<br>**Reverse:** 5'- AGGTTTTGGTGGAGGATATAG -3'
**STAT3**	**Forward:** 5'- GATTCATTGATGCAGTTTGG -3'<br>**Reverse:** 5'- GGTGTCTCAGTCATACTCTG -3'
**OPN**	**Forward:** 5'- CCAAGCGTGGAAACACACAGCC -3'<br>**Reverse:** 5'- GGCTTTGGAACTCGCCTGACTG -3'
**OCN**	**Forward:** 5'- ACCCTCTCTCTGCTCACTCTGC -3'<br>**Reverse:** 5'- TATTCACCACCTTACTGCCCTCC -3'
**BSP**	**Forward:** 5'- AAGCATGCCTACTTTTATCC -3'<br>**Reverse:** 5'- CTTCATTTGAAGTCTCCTCTTC -3'
**ALP**	**Forward:** 5'- ATGTCTGGAACCGCACTGAAC -3'<br>**Reverse:** 5'- AGCCTTTGGGATTCTTTGTCAG -3'

**RUNX2**	**Forward:** 5’- CAGTATGAGAGTAGGTGTCCCGC -3’<br>**Reverse:** 5’- AAGAGGGGTAAGACTGGTCATAGG -3’
**BMP2**	**Forward:** 5’- ATGGGTTTGTGGTGGAAGTG -3’<br>**Reverse:** 5’- AGTTCAGGTGATCAGCCAGG -3’
**BMP6**	**Forward:** 5’- AAAAGGAGATCCTGTCGG -3’<br>**Reverse:** 5’- AGAGATCCAGCATGAAGAG -3’
**COL1A1**	**Forward:** 5’- CCCCAAGGAGAAGAAGCATG -3’<br>**Reverse:** 5’- GAATCGACTGTTGCCTTCGC -3’
**Wnt5a**	**Forward:** 5’- CTTCCAAGTTCTTCCTAATGG -3’<br>**Reverse:** 5’- TTAGGGACCACCAAGAATTAG -3’
**Wnt3a**	**Forward:** 5’- AACACAGCAGCTTAATGAC -3’<br>**Reverse:** 5’-ATCTCCACGTAGTTCCTG -3’
**Wnt10b**	**Forward:** 5’- GTAATCACGACATGGACTTTG -3’<br>**Reverse:** 5’- CTCCAACAGGTCTTGAATTG -3’
**Axin2**	**Forward:** 5’- GAATACGAAAGGCACAGAAC -3’<br>**Reverse:** 5’- CTTATGCTTTGGGTACTATGG -3’
**β-catenin**	**Forward:** 5’- CATTGGTGCCCAGGGAGAAG -3’<br>**Reverse:** 5’- CCACCCATCTCATGCTCCATC -3’
**VEGFA**	**Forward:** 5’- CAATGATGAAGCCCTGGAGTG -3’<br>**Reverse:** 5’- GCTCATCTCTCCTATGTGCTGG -3’
**PDGFa**	**Forward:** 5’- CGACTCTTGGAGATAGACTC -3’<br>**Reverse:** 5’- CTTCCTCAATACTTCTCTTCC -3’
**PDGFb**	**Forward:** 5’- AGATCTCGCGGAACCTCATC -3’<br>**Reverse:** 5’- AGGGTCACTGTGGCCTTCTT -3’
**MMP9**	**Forward:** 5’- TACTTTGGAAACGCAAATGG -3’<br>**Reverse:** 5’- GTGTAGAGATTCTCACTGGG -3’
**CD31**	**Forward:** 5’- GATCTCCATCCTGTCGGGTAAC -3’<br>**Reverse:** 5’- GTGTCATTCACGGTTTCTTCGT -3’
**eNOS**	**Forward:** 5’- CAAGACCGATTACACGACATTGAGA -3’<br>**Reverse:** 5’- TGAGGACTTGTCCAAACACTCCAC -3’
**Ang-1**	**Forward:** 5’- TAACCTCGCCCTGCAAAGAG -3’<br>**Reverse:** 5’- CTGTATGCTTGCAGGTGGTGAT -3’
**α-SMA**	**Forward:** 5’- CAACCCCTATACAACCATCACAC -3’<br>**Reverse:** 5’- CCCAAACTGCTTGCGTAACC -3’
**GAPDH**	**Forward:** 5’- TCAGCAATGCCTCCTGCAC -3’<br>**Reverse:** 5’- TCTGGGTGGCAGTGATGGC -3’

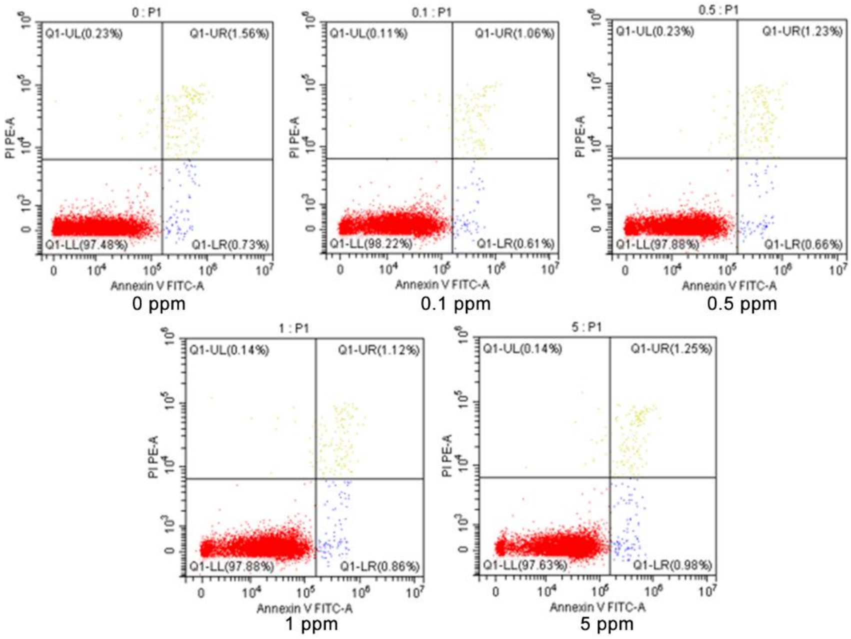

Supplementary Figure1. Cellular toxicity of Cobalt on PBMCs. Flow cytometry illustrates the apoptosis of PBMCs at different concentrations of $CoCl_2$ (0, 0.1, 0.5, 1, or 5 ppm) after the cobalt stimulation for 2 days.
